# Soluble urokinase plasminogen activator receptor and procalcitonin for risk stratification in patients with a suspected infection in the emergency department: a prospective cohort study

**DOI:** 10.1097/MEJ.0000000000001042

**Published:** 2023-06-08

**Authors:** Kirby Tong-Minh, Henrik Endeman, Christian Ramakers, Diederik Gommers, Eric van Gorp, Yuri van der Does

**Affiliations:** a Department of Emergency Medicine; b Department of Intensive Care; c Department of Clinical Chemistry; d Department of Internal Medicine; e Department of Viroscience, Erasmus University Medical Center, Rotterdam, the Netherlands

**Keywords:** biomarkers, NEWS2, procalcitonin, qSOFA, suPAR

## Abstract

**Background and importance:**

Early identification of patients at risk of clinical deterioration may improve prognosis of infected patients in the emergency department (ED). Combining clinical scoring systems with biomarkers may result in a more accurate prediction of mortality than a clinical scoring system or biomarker alone.

**Objective:**

The objective of this study is to investigate the performance of the combination of National Early Warning Score-2 (NEWS2) and quick Sequential Organ Failure Assessment (qSOFA) score with soluble urokinase plasminogen activator receptor (suPAR) and procalcitonin to predict 30-day mortality in patients with a suspected infection in the ED.

**Design, settings and participants:**

This was a single-center prospective observational study, conducted in the Netherlands. Patients with suspected infection in the ED were included in this study and followed-up for 30 days. The primary outcome of this study was all cause 30-day mortality. The association between suPAR and procalcitonin with mortality was assessed in subgroups of patients with low and high qSOFA (<1 and ≥1) and low and high NEWS2 (<7 and ≥7).

**Main results:**

Between March 2019 and December 2020, 958 patients were included. A total of 43 (4.5%) patients died within 30 days after ED visit. A suPAR ≥ 6 ng/ml was associated with an increased mortality risk: 5.5 vs. 0.9% (*P* < 0.01) in patients with qSOFA = 0 and 10.7 vs. 2.1% (*P* = 0.02) in patients with qSOFA ≥ 1. There was also an association between procalcitonin ≥0.25 ng/ml and mortality: 5.5 vs. 1.9% (*P* = 0.02) for qSOFA = 0 and 11.9 vs. 4.1% (*P* = 0.03) for qSOFA ≥ 1. Similar associations were found within patients with a NEWS < 7 (5.9 vs. 1.2% for suPAR and 7.0 vs. 1.7% for procalcitonin, *P* < 0.001).

**Conclusion:**

In this prospective cohort study, suPAR and procalcitonin were associated with increased mortality in patients with either a low or high qSOFA and patients with low NEWS2.

## Background

In the emergency department (ED), it is important to identify patients at risk of developing sepsis early. These patients require hospital admission and may require more intensive monitoring or treatment, while on the other hand, patients with a mild infection may be discharged early from the ED [1–3].

Physicians determine the severity of disease by clinical assessment with the addition of vital signs and laboratory parameters. This clinical assessment relies on the experience of the physician, which makes it subjective to the individual physician and may vary between different physicians [4]. Therefore, clinical scoring systems have been developed to predict disease severity objectively. Two commonly used clinical scoring systems are the National Early Warning Score-2 (NEWS2) and quick Sequential Organ Failure Assessment (qSOFA) score [5–7]. The benefit of these clinical scoring systems is that they only require vital parameters, which are routinely available in the ED and are not subjective to the level of training or experience of the clinician. Serum biomarkers can also aid in risk stratification in the ED. Different biomarkers are routinely used, including C-reactive protein and white blood cell count [8]. In patients with a suspected infection, procalcitonin can be used to identify bacterial infections. In the ED, elevated procalcitonin levels are also associated with severe infections [9]. A more general biomarker of disease severity is soluble urokinase plasminogen activator receptor (suPAR). suPAR is a protein expressed on endothelial and immune cells and suPAR levels are associated with the level of immune activation. Therefore, suPAR levels are predictive of disease severity in a general ED population, regardless of the reason of ED visit [10].

Despite these different methods that are available for risk stratification in the ED, they are often not standard-of-care. Clinical scoring systems and biomarkers alone may not be reliable enough to be used for clinical decision making. However, combining clinical scoring systems with biomarkers may result in a more accurate prediction of mortality than a clinical scoring system or biomarker alone [11].

The goal of this study is to investigate the performance of the combination of NEWS2 and qSOFA score with suPAR and procalcitonin on risk stratification on 30-day mortality in patients with a suspected infection in the ED.

## Methods

This study was a prospective observational study, registered as the FORESEEN study under registration number NL7576 in the Dutch Trial Registry. The FORESEEN study was conducted in the ED of Erasmus University Medical Center, an academic hospital with 40 000 ED visits yearly. This study with protocol name ‘Biomarkers for predicting disease severity in ED patients with suspected infection (FORESEEN)’ was approved by the Medical Ethical Committee Erasmus MC under number MEC-2018-1632 on 7 March 2019. Written informed consent was obtained from all included patients. All study procedures were performed in accordance with the ethical standards of the responsible committee on human experimentation and with the Helsinki Declaration of 1975. This study is reported according to the STrengthening the Reporting of OBservational studies in Epidemiology guidelines.

### Inclusion and exclusion criteria

Adult (aged 18 years or older) patients visiting the ED with suspected infection who gave informed written consent were included. Infection was suspected if a diagnosis (cultures, PCR) was ordered by the attending physician or if a curative antibiotic treatment was prescribed to the patient. Patients were excluded if they did not speak Dutch or had no permanent place of residence.

### Sample size calculation

Due to availability of research personnel to enroll patients in this study, we used a convenience sample of patients visiting the ED between March 2019 and December 2020. Based on previous studies in this ED, we aimed to enroll 1000 patients during this period.

### Biomarker measurements

After obtaining informed consent, 3 ml venous blood was drawn in a heparin-lithium tube (BD Barricor, Franklin Lakes, New Jersey, USA). The blood samples were centrifuged, aliquoted and frozen at −8 °C degrees. Procalcitonin and suPAR were measured in batch after enrollment was finished. Procalcitonin was measured using E801 Elecsys BRAHMS procalcitonin reagent on a COBAS 8000 (Roche Diagnostics, Basel, Switzerland) analyzer. suPAR was measured using a turbidimetric assay (Virogates, Birkerød, Denmark) on a COBAS 6000 (Roche Diagnostics, Switzerland) analyzer. The results of procalcitonin and suPAR were not available to the treating physicians and laboratory personnel were blinded to the outcomes of the study.

### Data collection

Patient data were collected during the ED visit and consisted of demographic data, comorbidities, vital signs during triage, routine laboratory tests and microbiological cultures. Laboratory and microbiological tests were only available when ordered by the treating physician. A patient record and questionnaire follow-up took place after 30 days where data on duration of hospital stay, ICU admission and mortality was recorded. To collect this follow-up data, patients received a questionnaire by email. In case the patient died within 30 days, the general practitioner of the patient was contacted for follow-up data.

### Outcomes

The primary outcome of this study was all-cause 30-day mortality. The secondary outcome of the study was hospital admission after ED visit.

### Statistical analysis

Normally distributed variables were reported as mean with SD and non-normally distributed variables as median with interquartile range (IQR). Multiple imputation was used for handling missing data. We performed the analyses in the original and imputed dataset and tested for differences between these datasets. If no significant differences were detected, we presented the data from the imputed dataset.

Differences in dichotomous variables between survivors and non-survivors were analyzed with chi-square tests. Differences in continuous variables were analyzed using an independent sample *T*-test for normally distributed data and a Mann–Whitney *U*-test for non-normally distributed data.

### Primary analysis

For the primary analysis, we used stepwise risk stratification providing the mortality risk before and after using the NEWS2 score and qSOFA score, followed by the final mortality risk after combining this mortality risk with the mortality risk at different levels of suPAR and procalcitonin. To create a decision aid for clinicians that are applicable in clinical practice, we transformed the clinical scoring systems and biomarkers into a binary variable, creating a low-risk and high-risk category for both clinical scoring systems and biomarkers. For the NEWS2 score we used a cut-off of seven points, which is higher than the commonly used threshold of five points in the hospital wards, because ED patients often have multiple abnormal vital signs during the acute phase of their illness. For the qSOFA score we used a cut-off of one point [5]. For suPAR we used a cut-off value of 6.0 ng/ml as suggested by previous studies in the ED [12]. For procalcitonin, we used a cut-off value of 0.25 ng/ml [13].

We calculated the 30-day mortality all-cause mortality risk, followed by the mortality risk in the low and high categories of NEWS2 and qSOFA with corresponding 95% confidence interval (CI). We then combined these risk categories with the low- and high-risk categories of suPAR and procalcitonin, calculating the mortality risk in these risk categories combined with the low and high categories of NEWS2 and qSOFA. Finally, we tested the risk difference between the low and high suPAR and procalcitonin in the low and high NEWS2 and qSOFA groups.

### Secondary analyses

Additionally, we used stepwise risk stratification with hospital admission as secondary outcome.

Furthermore, as a secondary analysis, we used univariate logistic regression analysis and area under the curve (AUC) analysis to investigate the prognostic ability of biomarkers suPAR and procalcitonin and clinical scoring systems NEWS2 and qSOFA on 30-day mortality and hospital admission alone. Finally we used multivariate regression analysis and AUC analysis to calculate the AUC of the combinations of NEWS2 with suPAR or procalcitonin and qSOFA with NEWS2 or procalcitonin. For this calculation, the AUC of NEWS2 and qSOFA was calculated per point increase of these scores.

We used ‘R’ version 4.0.1 (The R Foundation, Vienna, Austria) for statistical analyses. The ‘MICE’ package (MICE 3.13, Leiden, The Netherlands) was used for multiple imputation, using the first imputation after 50 iterations.

## Results

**Fig. 1:**
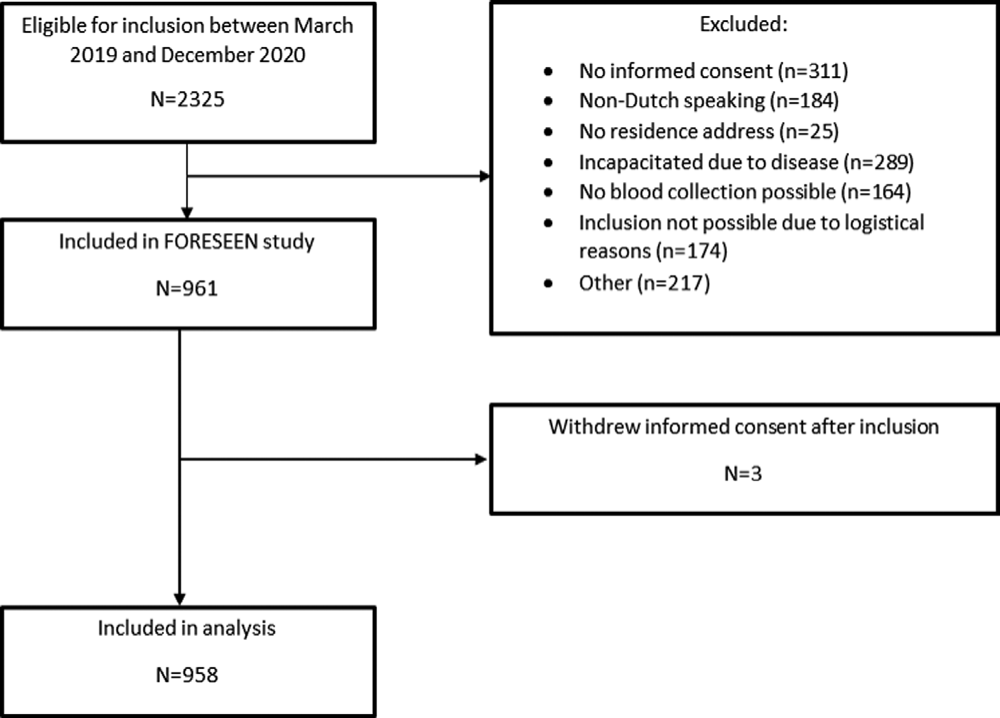
Flowchart of included patients.

The FORESEEN study was conducted between 15 March 2019 and 27 November 2020. A total of 2325 patients were screened for eligibility, of which 958 were included in the FORESEEN study (Fig. 1). An overview of missing data is shown in Supplementary File 1, Supplemental digital content, http://links.lww.com/EJEM/A376. We found no difference in the imputed dataset compared to the original dataset and performed all analyses in the imputed dataset.

A total of 43 (4.5%) patients died within 30 days after ED visit. Table 1 shows the baseline characteristics between survivors and non-survivors. A total of 543 (56.7%) patients had a bacterial infection, 158 (16.5%) patients had a viral infection, 38 (4.0%) patients had a combined bacterial and viral infection and 295 (30.8%) patients were diagnosed with a non-infectious alternative diagnosis. The most common origin of infection were pulmonary infections in 384 (40.1%) patients, followed by abdominal infections in 203 (21.2%) patients, urogenital infections in 137 (14.3%) patients and skin infections in 71 (7.4%) patients.

**Table 1:** Baseline characteristics with differences between survivors and non-survivors.

	All patients	Survivor	Non-survivor	
Patient characteristics	*n* = 958	*n* = 915	*n* = 43	*P*-value
Demographic data				
Age (years)	60.0 (47.0–69.0)	60.0 (46.0–69.0)	65.0 (60.0–70.5)	0.004
Gender: male	545 (56.9%)	519 (56.7%)	26 (60.5%)	0.744
Comorbidity: Cardiovascular disease	360 (37.6%)	337 (36.8%)	23 (53.5%)	0.041
Comorbidity: Pulmonary disease	262 (27.3%)	246 (26.9%)	16 (37.2%)	0.190
Comorbidity: Diabetes mellitus	175 (18.3%)	161 (17.6%)	14 (32.6%)	0.023
Comorbidity: Renal disease	152 (15.9%)	142 (15.5%)	10 (23.3%)	0.253
Comorbidity: Liver disease	111 (11.6%)	106 (11.6%)	5 (11.6%)	1
Comorbidity: Malignancy	315 (32.9%)	286 (31.3%)	29 (67.4%)	<0.001
Comorbidity: Auto-immune diseases	113 (11.8%)	112 (12.2%)	1 (2.3%)	0.084
Comorbidity: Immunodeficiency	43 (4.5%)	42 (4.6%)	1 (2.3%)	0.746
Comorbidity: Transplantation	179 (18.7%)	172 (18.8%)	7 (16.3%)	0.831
Vital parameters				
Heart rate (beats per minute)	94.6 (±19.0)	94.5 (±18.7)	97.0 (±25.2)	0.539
Respiratory rate (per minute)	18.0 (±6.00)	18.0 (±6.00)	20.0 (±8.00)	0.014
Oxygen saturation (%)	96.0 (95.0–98.0)	96.0 (95.0–98.0)	95.0 (93.5–97.0)	0.010
Supplemental oxygen	121 (12.6%)	106 (11.6%)	15 (34.9%)	<0.001
Systolic blood pressure (mmHg)	134 (±23.3)	135 (±22.7)	122 (±31.2)	0.008
Diastolic blood pressure (mmHg)	80.1 (±15.4)	80.6 (±15.1)	70.1 (±18.3)	<0.001
Temperature (℃)	37.5 (±0.919)	37.5 (±0.920)	37.4 (±0.890)	0.976
Glasgow Coma Score	15 (15–15)	15 (15–15)	15 (15–15)	1
Laboratory testing				
CRP (mg/l)	60.0 (16.0–128)	56.0 (15.0–122)	126 (79.0–245)	<0.001
WBC	9.35 (6.50–13.4)	9.30 (6.50–13.3)	12.2 (8.55–17.7)	0.008
Procalcitonin (ng/ml)	0.170 (0.070–0.580)	0.160 (0.060–0.545)	0.580 (0.170–3.32)	<0.001
suPAR (ng/ml)	6.40 (4.40–9.60)	6.30 (4.40–9.10)	11.50 (8.05–13.9)	<0.001
Clinical scoring systems				
NEWS2	2 (1–4)	2 (1–4)	4 (2–7)	<0.001
qSOFA	0 (0–1)	0 (0–1)	0 (0–1)	0.005

CRP, C-reactive protein; NEWS2, National Early Warning Score 2; qSOFA, quick Sequential Organ Failure Assessment; suPAR, soluble urokinase plasminogen activator receptor; WBC, white blood cell count.

The median NEWS2 and qSOFA score were 2 (IQR: 1–3) and 0 (IQR: 0–1) respectively. Both NEWS2 and qSOFA score were significantly higher in non-survivors compared to survivors (*P* < 0.001 and *P* = 0.005 respectively). The median suPAR value was 6.4 ng/ml (IQR: 4.40–9.60 ng/ml). The median suPAR was significantly higher in non-survivors than in survivors (6.30 vs. 11.50 ng/ml, *P* < 0.001). The median procalcitonin value was 0.17 ng/ml (IQR: 0.070–0.580 ng/ml). Procalcitonin was also significantly higher in non-survivors compared to survivors (0.160 vs. 0.580 ng/ml, *P* < 0.001).

The all-cause 30-day mortality risk was 4.5% (95% CI: 3.3–6.0%). After transforming the NEWS2 score into a low-risk and high-risk category with a cut-off of seven points, 880 (91.9%) patients were categorized as low risk and 78 (8.1%) patients as high risk. For the qSOFA score, 695 (72.5%) patients were categorized as low risk and 263 (27.4%) were categorized as high risk, using a cut-off value of one point. When transforming suPAR in a low- and high-risk category using a cut-off value of 6.0 ng/ml, 424 (44.3%) patients were categorized as low risk and 534 (55.7%) patients as high risk. Transforming procalcitonin in a low- and high-risk category using a cut-off value of 0.25 ng/ml resulted in 569 (59.4%) patients categorized as low risk and 389 (40.6%) patients as high risk.

### Risk stratification on 30-day mortality

The combinations of clinical scoring system and biomarker categories and corresponding 30-day mortality risk are shown in Table 2.

**Table 2:** Mortality risk of combinations of NEWS2 and qSOFA with suPAR and procalcitonin.

Clinical scoring system	suPAR low (<6.0 ng/ml)	suPAR high (≥6.0 ng/ml)	Risk difference	PCT low (<0.25 ng/ml)	PCT high (≥0.25 ng/ml)	Risk difference	All patients
NEWS2 low (<7)	1.2% (0.4–2.8%) 5/409	5.9% (4.0–8.5%) 28/471	4.7% (2.3–7.1%)	1.7% (0.8–3.2%) 9/538	7.0% (4.5–10.3%) 24/342	5.3% (2.4–8.3%)	3.8% (2.6–5.2%) 33/880
NEWS2 high (≥7)	0% (0–21.8%) 0/15	15.9% (7.9% - 27.3%) 10/63	15.9% (6.8–24.9%)	16.1% (5.4–33.7%) 5/31	10.6% (3.5–23.1%) 5/47	−5.5% (-21.1–10.2%)	12.8% (6.3–22.3%) 10/78
qSOFA low (<1)	0.9% (0.2–2.6%) 3/330	5.5% (3.4–8.3%) 20/365	4.6% (2.0–7.1%)	1.9% (0.8–3.7%) 8/424	5.5% (3.1–8.9%) 15/271	3.6% (0.6–6.7%)	3.3% (2.1–4.9%) 23/695
qSOFA high (≥1)	2.1% (0.3–7.5%) 2/94	10.7% (6.4–16.3) 18/169	8.5% (3.0–14.0%)	4.1% (1.5–8.8%) 6/145	11.9% (6.6–19.1%) 14/118	7.7% (1.1–14.4%)	7.6% (4.7–11.5%) 20/263

Mortality risk is presented with 95% confidence interval and absolute count.

NEWS2, National Early Warning Score 2; qSOFA, quick Sequential Organ Failure Assessment; suPAR, soluble urokinase plasminogen activator receptor.

A suPAR ≥ 6 ng/ml was associated with an increased mortality risk: 5.5 vs. 0.9% (difference 4.6, 95% CI: 2.0–7.1%) in patients with qSOFA = 0 and 10.7 vs. 2.1% (difference 8.5, 95% CI 3.0–14.0%) in patients with qSOFA ≥ 1. There was also an association between procalcitonin ≥0.25 ng/ml and mortality: 5.5 vs. 1.9% in patients with qSOFA = 0 (difference 3.6, 95% CI: 0.6–6.7%) and 11.9 vs. 4.1% (difference 7.7, 95% CI: 1.1–14.4%) in patients with qSOFA ≥ 1. Similar associations were found within patients with a NEWS < 7 : 5.9 vs. 1.2% for suPAR (difference 4.7, 95% CI: 2.3–7.1%) and 7.0 vs. 1.7% for procalcitonin (difference 5.3, 95% CI: 2.4–8.3%).

There was no statistical difference within patients with a NEWS2 ≥ 7.

**Fig. 2:**
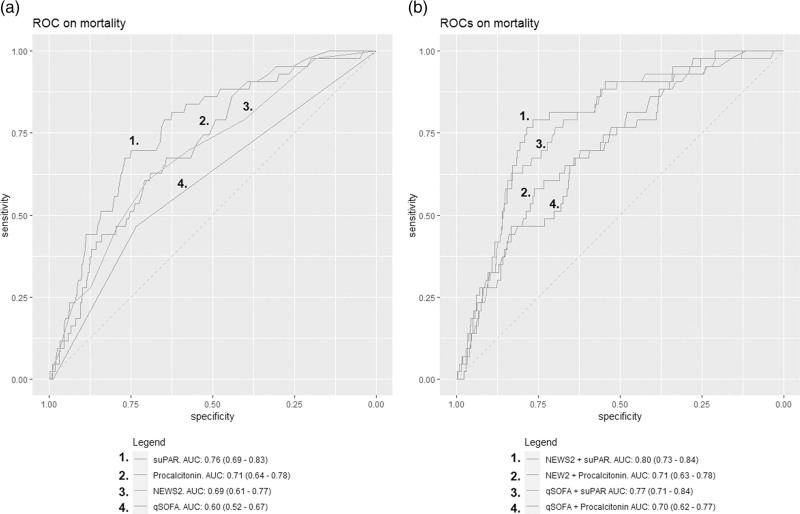
Receiver operating characteristics and corresponding area under the curve with 95% confidence interval of (a) soluble urokinase plasminogen activator receptor, procalcitonin, National Early Warning Score 2 and quick Sequential Organ Failure Assessment alone and (b) National Early Warning Score 2 and quick Sequential Organ Failure Assessment combined with soluble urokinase plasminogen activator receptor and procalcitonin.

In univariate logistic regression analysis, the odds ratio (OR) on 30-day mortality of suPAR, procalcitonin, NEWS2 and qSOFA were 1.19 (95% CI: 1.13–1.26), 1.02 (95% CI: 0.99–1.03), 1.28 (95% CI: 1.16–1.42) and 2.32 (95% CI: 1.36–3.91) respectively. The receiver operating characteristics and corresponding AUCs of suPAR, procalcitonin, NEWS2 and qSOFA are shown in Fig. 2a. The AUCs of suPAR and procalcitonin combined with the NEWS2 and qSOFA score are shown in Fig. 2b.

### Risk stratification on hospital admission

The risk of hospital admission in the entire cohort was 63.3% (95% CI: 60.2–66.4%). The risk of admission stratified by the different risk categories of NEWS2 and qSOFA combined with suPAR and procalcitonin are shown in Supplementary File 2, Supplemental digital content, http://links.lww.com/EJEM/A376. A high suPAR or procalcitonin were associated with an increased admission risk compared to a low suPAR or procalcitonin in all NEWS2 and qSOFA categories, except for patients with a high NEWS2 score. In univariate logistic regression analysis, the OR on hospital admission of suPAR, procalcitonin, NEWS2 and qSOFA were 1.22 (95% CI: 1.17–1.28), 1.26 (95% CI: 1.15–1.42), 1.30 (95% CI: 1.22–1.39) and 1.99 (95% CI: 1.49–2.68) respectively. The AUCs were 0.70 (95% CI: 0.67–0.74), 0.71 (95% CI: 0.67–0.74), 0.65 (95% CI: 0.62–0.68) and 0.57 (95% CI: 0.54–0.59) respectively.

## Discussion

In this study, we investigated if the combination of clinical scoring systems NEWS2 and qSOFA score with biomarkers suPAR and procalcitonin are more accurate in 30-day mortality risk stratification than the clinical scoring systems and biomarkers alone in patients with a suspected infection in the ED. We found that a high suPAR and procalcitonin were associated with an increased mortality risk compared to a low suPAR and procalcitonin in most patients with either a low or high NEWS2 or qSOFA. This finding shows that combining suPAR and procalcitonin to clinical scoring systems NEWS2 and qSOFA can result in a different mortality risk. Patients classified as low risk by NEWS2 and qSOFA still have a high mortality risk when they have a high suPAR or NEWS2. This shows that these biomarkers may detect clinical deterioration before vital signs turn abnormal and physicians should consider monitoring these patients even when vital signs are normal. On the other hand, when patients have a high NEWS2 with a low suPAR or a high qSOFA with either a low suPAR or procalcitonin, the mortality risk is reduced and physicians may consider discharge from the ED. This finding supports other studies that showed that adding a biomarker often results in more accurate prediction of disease severity in patients with infectious diseases [11,14]. The biomarkers suPAR and procalcitonin individually did show an adequate prediction of 30-day mortality (AUCs of 0.76 and 0.71, respectively), which supports other studies that showed the benefit of using these biomarkers for assessing disease severity in the ED. Further studies are required to show if incorporating these biomarkers in clinical practice with either results in improved health outcomes. This could be done in an interventional study where the intervention arm includes suPAR and procalcitonin with a clear disposition advice based on the biomarker level compared to regular care without suPAR and procalcitonin the diagnostic workup.

In contrast to other biomarker or prediction model studies [8], we primarily presented the sequential post-test mortality and admission risks by combining the clinical scoring systems with biomarkers, instead of a multivariate regression analysis with corresponding AUC analysis or net benefit analysis [15]. Although statistically appropriate, the results provided by multivariate logistic regression analysis, AUC and net benefit analyses can be challenging to interpret by clinicians, because the results are too abstract to directly apply in clinical practice. The benefit of presenting a sequential post-test mortality risk is that it provides the actual mortality risk per category, which is intuitive to interpret [16]. In the univariate and AUC analysis, we found that the performance on 30-day mortality of suPAR and procalcitonin are in line with other studies [17,18]. The performance of qSOFA has been controversial with varying results in different studies. In our study, we found an AUC of 0.61 of qSOFA on 30-day mortality, which is considered too low to reliably predict mortality in the ED [7,19,20].

Procalcitonin is a biomarker primarily used for differentiating bacterial from viral infections [13]. However, a growing number of studies have shown that procalcitonin can also be used for predicting disease severity [17,18,21]. This is supported by our study, where we found an AUC of 0.72 of procalcitonin on 30-day mortality. suPAR showed the highest AUC on 30-day mortality in our study, which corresponds to other ED studies that investigated the predictive value of suPAR and also report AUCs between 0.80 and 0.84 [10,12].

The added value of combining qSOFA and NEWS2 with suPAR and procalcitonin was limited when used for risk stratification on hospital admission. Although we did find that a high suPAR or procalcitonin were associated with an increased admission risk in most patients compared to patients with a low suPAR or procalcitonin, the clinical consequence of this finding is not clear. The overall admission rate was 63.3% and the final risk category of all patients was 43% or higher. Therefore, it is difficult to translate these findings into a clinical disposition advice, because the final risk on hospital admission in all patients was too high to advise discharge from the ED.

## Limitations

A limitation of this study was the requirement of obtaining written informed consent from patients. As a result, patients that were already severely ill or unresponsive in the ED could not be included in this study. Therefore, the results of this study may not be valid for these patients. However, the ultimate goal of this study was to improve risk stratification in patients with a suspected infection. Patients who are already severely ill at ED usually don’t require additional tools for risk stratification because they will already show clear clinical signs of severe disease. Patients with an altered mental state were not able to give informed consent, resulting in a missing point in the qSOFA and NEWS2 score. To correct for this limitation we used a cut-off value of 1 for a high qSOFA score instead of 2 as suggested by previous studies. Because the NEWS2 score consists of seven other parameters, the impact of missing consciousness in this study may be limited. However, validation should be done in a cohort where patients with an altered mental state are also included.

Although this study included 958 patients, the number of fatalities was low. Therefore, these results require validation in a larger cohort, preferably in a multicenter setting also including non-academic hospitals to improve generalizability of the findings. Combining a two-category clinical scoring system with another two-category biomarker results in four combinations of final risk category, where the subgroups and absolute number of fatalities in some combinations of these groups were small. A single fatality could therefore result in a large difference in final risk category.

### Conclusion

In this prospective cohort study, a high level of suPAR and procalcitonin were significantly associated with an increased mortality risk compared in patients with either a low or high qSOFA and in patients with low NEWS2.

## Acknowledgements

K.T.-M. and Y.D. were involved in the conception or design of the manuscript. K.T.-M. and Y.D. performed the analysis and interpretation of the data. K.T.M. drafted the manuscript. K.T.-M., H.E., C.R., D.G., E.G., and Y.D. were involved in the critical revision of the manuscript and final approval of the manuscript. The datasets used and/or analyzed during the current study are available from the corresponding author on reasonable request.Virogates A/S provided the measurement kits for the purpose of this study. The manufacturer was not involved in the collection, analysis and reporting of the data.

### Conflicts of interest

There are no conflicts of interest.

## Supplementary Material

## References

[1] De Backer D Dorman T. Surviving sepsis guidelines: a continuous move toward better care of patients with sepsis. JAMA 2017; 317:807–808. 28114630 10.1001/jama.2017.0059

[2] Rivers E Nguyen B Havstad S Ressler J Muzzin A Knoblich B. Early goal-directed therapy in the treatment of severe sepsis and septic shock. N Engl J Med 2001; 345:1368–1377. 11794169 10.1056/NEJMoa010307

[3] de Groot B Jessen MK Nickel CH. The new 2021 Surviving Sepsis Guidelines: an emergency department perspective may be more effective. Eur J Emerg Med 2022; 29:5–6. 34932027 10.1097/MEJ.0000000000000898

[4] Pope I Burn H Ismail SA Harris T McCoy D. A qualitative study exploring the factors influencing admission to hospital from the emergency department. BMJ Open 2017; 7:e011543. 10.1136/bmjopen-2016-011543 PMC5577896 28851767

[5] Physicians RCo. National Early Warning Score (NEWS) 2: standardising the assessment of acute-illness severity in the NHS. Updated report of a working party. RCP; 2017. https://www.rcplondon.ac.uk/projects/outputs/national-early-warning-score-news-2. [Accessed 16 August 2022]

[6] Singer M Deutschman CS Seymour CW Shankar-Hari M Annane D Bauer M. The third international consensus definitions for sepsis and septic shock (Sepsis-3). JAMA 2016; 315:801–810. 26903338 10.1001/jama.2016.0287 PMC4968574

[7] Zonneveld L van Wijk RJ Olgers TJ Bouma HR Ter Maaten JC. Prognostic value of serial score measurements of the national early warning score, the quick sequential organ failure assessment and the systemic inflammatory response syndrome to predict clinical outcome in early sepsis. Eur J Emerg Med 2022; 29:348–356. 36062434 10.1097/MEJ.0000000000000924 PMC9432814

[8] Pierrakos C Velissaris D Bisdorff M Marshall JC Vincent JL. Biomarkers of sepsis: time for a reappraisal. Crit Care 2020; 24:287. 32503670 10.1186/s13054-020-02993-5 PMC7273821

[9] Gautam S Cohen AJ Stahl Y Valda Toro P Young GM Datta R. Severe respiratory viral infection induces procalcitonin in the absence of bacterial pneumonia. Thorax 2020; 75:974–981. 32826284 10.1136/thoraxjnl-2020-214896

[10] Rasmussen LJ Ladelund S Haupt TH Ellekilde G Poulsen JH Iversen K. Soluble urokinase plasminogen activator receptor (suPAR) in acute care: a strong marker of disease presence and severity, readmission and mortality. A retrospective cohort study. Emerg Med J 2016; 33:769–775. 27590986 10.1136/emermed-2015-205444 PMC5136705

[11] Tong-Minh K Welten I Endeman H Hagenaars T Ramakers C Gommers D. Predicting mortality in adult patients with sepsis in the emergency department by using combinations of biomarkers and clinical scoring systems: a systematic review. BMC Emerg Med 2021; 21:70. 34120605 10.1186/s12873-021-00461-z PMC8201689

[12] Santeri S Peter AA Kristiina N Jesper EO Harri H. suPAR cut-offs for stratification of low, medium, and high-risk acute medical patients in the emergency department. BMC Emerg Med 2021; 21:149. 34844557 10.1186/s12873-021-00544-x PMC8628287

[13] Hoeboer SH van der Geest PJ Nieboer D Groeneveld AB. The diagnostic accuracy of procalcitonin for bacteraemia: a systematic review and meta-analysis. Clin Microbiol Infect 2015; 21:474–481. 25726038 10.1016/j.cmi.2014.12.026

[14] Rasmussen LJH Ladelund S Haupt TH Ellekilde GE Eugen-Olsen J Andersen O. Combining National Early Warning Score with soluble urokinase plasminogen activator receptor (suPAR) improves risk prediction in acute medical patients: a registry-based cohort study. Crit Care Med 2018; 46:1961–1968. 30247244 10.1097/CCM.0000000000003441 PMC6250248

[15] Vickers AJ Van Calster B Steyerberg EW. Net benefit approaches to the evaluation of prediction models, molecular markers, and diagnostic tests. BMJ 2016; 352:i6. 26810254 10.1136/bmj.i6 PMC4724785

[16] Cei F Fenu P Sole C Mumoli N Cei M. A quick modified early warning score for triaging medical patients at admission. Eur J Emerg Med 2022; 29:80–81. 34932034 10.1097/MEJ.0000000000000844

[17] Kim SJ Hwang SO Kim YW Lee JH Cha KC. Procalcitonin as a diagnostic marker for sepsis/septic shock in the emergency department; a study based on Sepsis-3 definition. Am J Emerg Med 2019; 37:272–276. 29861371 10.1016/j.ajem.2018.05.047

[18] Tong-Minh K van der Does Y Engelen S de Jong E Ramakers C Gommers D. High procalcitonin levels associated with increased intensive care unit admission and mortality in patients with a COVID-19 infection in the emergency department. BMC Infect Dis 2022; 22:165. 35189826 10.1186/s12879-022-07144-5 PMC8860271

[19] Lo RSL Leung LY Brabrand M Yeung CY Chan SY Lam CCY. qSOFA is a poor predictor of short-term mortality in all patients: a systematic review of 410,000 patients. J Clin Med 2019; 8:61. 30626160 10.3390/jcm8010061 PMC6351955

[20] Ruan H Ke D Liao D. Prognostic accuracy of qSOFA and SIRS for mortality in the emergency department: a meta-analysis and systematic review of prospective studies. Emerg Med Int 2022; 2022:1802707. 35572161 10.1155/2022/1802707 PMC9098353

[21] Heer RS Mandal AKJ Szawarski P Missouris CG. Procalcitonin is a biomarker for disease severity rather than bacterial co-infection in COVID-19. Eur J Emerg Med 2022; 29:315. 34560701 10.1097/MEJ.0000000000000882 PMC9241554

