# Supplementary material for: Soluble urokinase plasminogen activator receptor and procalcitonin for risk stratification in patients with a suspected infection in the emergency department: a prospective cohort study
Source: Eur J Emerg Med. 2023 Jun 8;30(5):324–30. doi: 10.1097/MEJ.0000000000001042 (PMC10467805; doi:10.1097/MEJ.0000000000001042)
Supplement: Supplementary file 1 [file ejem-30-324-s001.pdf]

Supplementary files for:

Combining clinical scoring systems with biomarkers for risk stratification in patients with a suspected infection in the emergency department: a prospective observational cohort study.

Supplementary File 1

Variable	Number of patients missing	Percentage missing
Heartrate	26	2.7%
Systolic blood pressure	15	1.6%
Diastolic blood pressure	16	1.7%
Temperature	15	1.6%
Respiratory Rate	158	16.5%
Peripheral oxygen saturation	18	1.9%
CRP	20	2.1%
WBC	16	1.7%
Procalcitonin	28	2.9%
suPAR	59	6.2%

Supplementary Table 1. Overview of missing data. Missing data was imputed by multiple imputation. CRP: C-reactive protein. WBC: White blood cell count. suPAR: soluble urokinase plasminogen activator receptor

Supplementary File 2

Clinical scoring system	suPAR low (<6.0 ng/mL)	suPAR high (≥6.0 ng/mL)	Risk difference	PCT low (<0.25 ng/mL)	PCT high (≥0.25 ng/mL)	Risk difference	All patients
**NEWS2 low (<7)**	45.7% (40.8 – 50.7%) 187/409	73.9% (69.7 – 77.8%) 348/471	28.2% (21.9 – 34.4%)	50.0% (45.7– 54.3%) 269/538	77.8% (73.0 – 82.1%) 266/342	27.8% (21.7 – 33.9%%)	60.8% (57.5 – 64.0%) 535/880
**NEWS2 high (≥7**)	86.7% (59.5 – 98.3%) 13/15	93.7% (84.5% - 98.2%) 59/63	7.0% (-11.2 – 25.2%)	87.1% (70.2 – 96.3%) 27/31	95.7% (85.5 – 99.5%) 45/47	8.6% (-4.5 – 21.8%)	92.3% (84.0 – 97.1%) 72/78
**qSOFA low (<1)**	43.0% (37.6 – 48.6%) 142/330	73.4% (68.6 – 77.9%) 268/365	30.4% (23.4 – 37.4%)	47.6% (42.8 – 52.5%) 202/424	76.8% (71.3 – 81.6%) 208/271	29.1% (22.2 – 36.0%)	59.0% (55.2 – 62.7%) 410/695
**qSOFA high (≥1)**	61.7% (51.1 – 71.5%) 58/94	82.2% (75.6 – 87.7%) 139/169	20.5% (9.2 – 31.9%)	64.8% (56.5 – 72.6%) 94/145	87.3% (80.0 – 92.7%) 103/118	22.5% (12.6 – 32.3%)	7.6% (4.7 – 11.5%) 20/263

Supplementary table 3. Admission risk of combinations of NEWS2 and qSOFA with suPAR and procalcitonin. Admission risk is presented with 95% confidence interval and absolute count.

Procalcitonin. suPAR: soluble urokinase plasminogen activator receptor. NEWS2: National early warning score 2. qSOFA: Quick sequential organ failure assessment.
